# Accurate Inverse Design of Broadband Solar Metamaterial Absorbers via Joint Forward–Inverse Deep Learning

**DOI:** 10.3390/nano16050297

**Published:** 2026-02-26

**Authors:** Qihang Wu, Zhiming Deng, Cong Zeng, Haoyuan Cai

**Affiliations:** 1 College of Ocean Information Engineering, Jimei University, Xiamen 361021, China; 202312854006@jmu.edu.cn (Q.W.); ycdeng@jmu.edu.cn (Z.D.); 202312854011@jmu.edu.cn (C.Z.); 2 Fujian Provincial Key Laboratory of Oceanic Information Perception and Intelligent Processing, School of Ocean Information Engineering, Jimei University, Xiamen 361021, China

**Keywords:** deep learning, metamaterial absorber, solar–thermal conversion, hyperbolic metamaterial, inverse design

## Abstract

The design of broadband, high-efficiency solar absorbers remains challenging due to the complex and ill-posed inverse mapping from the target optical responses to the physical structures in inverse design optimization. To address this, we propose a joint forward–inverse deep learning framework that enables the rapid and accurate optimization of multilayer metamaterial absorbers. This method integrates an inverse network based on a Modified Swin Transformer with a Multilayer Perceptron forward proxy and performs end-to-end training in a consistency-driven cycle. This strategy reduces the one-to-many ambiguity in inverse design and improves the prediction accuracy, with normalized test mean squared errors of 7.2 × 10^−5^ (inverse) and 6.8 × 10^−5^ (forward). Using this framework, we optimized an absorber comprising W/SiO_2_ hyperbolic metamaterial stacks and TiO_2_/SiO_2_ anti-reflection coatings, achieving 97.4% average absorptivity across the 400–1750 nm solar spectrum, along with polarization insensitivity and robust wide-angle performance up to 60° incidence. The outdoor solar heating tests showed that the fabricated absorber reaches a peak temperature of 86.3 °C under natural sunlight, with an irradiance peak of about 850 W/m^2^ at noon. This work shows that combining forward and reverse deep learning provides a powerful and scalable paradigm for accelerating the intelligent design of high-performance solar thermal metamaterials.

## 1. Introduction

As solar energy is increasingly used for applications like desalination and power generation, efficient solar-to-thermal conversion becomes critical [1,2]. Solar absorbers with near-perfect absorption over the visible to near-infrared spectrum are key to maximizing light-to-heat conversion efficiency. Therefore, designing and fabricating such high-performance broadband absorbers is essential for advancing solar thermal technologies [3,4,5].

Numerous structural configurations have been explored to achieve the broadband, high-absorption performance of solar absorbers. These include periodically arranged tungsten pyramids, hyperbolic metamaterials (HMMs) based on nanoporous tungsten–silica layers, along with sawtooth and various other periodic nanostructures [6,7,8]. For instance, Chen et al. developed an innovative nanowire-based composite structure, the core of which is gold nanowires evenly distributed in a truncated pyramid-shaped silica framework. This unique micro-nano architecture successfully achieves a broadband light absorption performance of over 90% in the ultraviolet to visible light band from 200 to 600 nm [9]. Li et al. successfully developed an ultra-broadband perfect absorber based on a periodically stacked structure of metal–insulator composite films. This device exhibits an absorption efficiency of over 90% in the near-infrared to mid-infrared band (570–3539 nm). This breakthrough achievement has opened up new possibilities for applications in optoelectronic devices and energy conversion fields [10]. Yuan et al. designed an annular structure solar absorber composed of alternating multiple layers of Ti and SiO_2_. This structure exhibits excellent ultra-broadband light absorption characteristics, with an average absorption rate of up to 98.03% in the full spectral range from 280 to 4000 nm, and even under simulated AM1.5 standard sunlight conditions, its average absorption efficiency can reach 97.66% [11].

Nevertheless, these patterned nanostructures generally rely on sophisticated nanofabrication processes [12]. By contrast, multilayer structures have emerged as promising alternatives to overcome this limitation, owing to their relatively simple fabrication procedures and lithography-free nature [13]. Among the various multilayer designs, stacked dielectric–metal architectures have recently attracted considerable attention for realizing broadband absorption. Most of the reported structures only consist of three or four layers and exhibit dominant absorption in the visible spectrum [14]. To extend the absorption bandwidth across the visible to near-infrared spectrum, it is usually necessary to increase the number of layers, which consequently introduces a large number of parameters requiring optimization [15]. Traditional manual optimization methods are not only time-consuming and labor-intensive, but also prone to failing to achieve optimal design performance [16].

The emergence of deep learning (DL) holds promise for solving the design bottleneck in the development and optimization of solar absorbers. In forward design, the structural parameters are input into a DL model, and the optical response can be quickly predicted without a time-consuming full-wave simulation. This speeds up the process of design evaluation and simplifies performance prediction during the development of solar absorbers [17]. For example, Gai et al. trained a forward deep neural network on the numerically generated spectra of a Ti/SiO_2_/Ti/SiO_2_/Fe five-layer metamaterial absorber and then used the network to efficiently search the parameter space, obtaining a design with an average absorptance of 98.70% from 170 to 900 nm and 99.27% in the visible range [18]. Almawgani et al. combined a weighted k-nearest-neighbor regression model with a long short-term memory (LSTM) time-series predictor to forecast the absorption spectra of a plus-shape slotted metamaterial solar absorber from a limited set of simulated data, achieving an R^2^ > 0.9 while reducing the required full-wave simulations and associated computational resources by about 50–60% [19].

Reverse design is different from the forward method. Starting from the target optical performance, such as ultra-wide spectral absorption, DL is used to predict the material composition, geometric structure or layer configuration [20]. Recent research results have shown that reverse design has high efficiency in solar absorber platforms. Ding et al. used an inverse model based on an artificial neural network (ANN) to map the target solar spectrum (approximately 280–4000 nm) to the geometric parameters of the metasurface absorber, and achieved an experimentally verified spectral-weighted solar absorptance of about 97% [21]. Xu et al. proposed a conditional generative adversarial network (CGAN). It extracts the free-form metasurface patterns from the optical responses to automatically search for unconventional nanostructures [22]. Guan et al., combining Bayesian optimization and scattering matrix modeling, explored the design space of ultrathin W/SiC nanocomposite solar absorbers, and quickly found the high-performance multilayer stacks with spectral selective absorption when there was not much prior computational space knowledge [23].

Building on these advances, researchers have begun exploring more powerful and flexible deep learning architectures to further enhance inverse design capability. Notably, Xu et al. integrated a Transformer with an encoder–decoder framework for the inverse design of multi-material thin-film solar absorbers [24]. Their model uniquely handles both discrete variables (e.g., material types, number of layers) and continuous variables (e.g., thicknesses), enabling end-to-end mapping from the target absorption spectra to complete, manufacturable multilayer structures. The experimental validation showed that this approach surpasses traditional optimization methods-—such as genetic algorithms and Bayesian optimization—in both design efficiency and convergence stability. Nevertheless, relying solely on inverse design is insufficient. Inverse models suffer from inherent flaws. The problem is ill-posed: one target spectrum may correspond to multiple structures, leading to non-unique solutions [25,26,27].

Based on the above discussion, we propose a broadband absorber composed of easily fabricable hyperbolic metamaterials (W/SiO_2_ planar stack) combined with a TiO_2_/SiO_2_ anti-reflective (AR) layer. For parameter optimization, we design a joint forward–inverse deep learning approach to achieve high optimization accuracy. The framework employs a tightly coupled three-stage training strategy that jointly optimizes an inverse model based on a Modified Swin Transformer (MST) and a forward model based on Multilayer Perceptron (MLP). Specifically, the inverse model maps the target absorption spectra to the structural parameters, while the forward model validates the physical feasibility of these parameters. A composite loss function—incorporating the reconstruction and regularization terms—enables cyclic feedback, which mitigates the ill-posed "one-to-many" mapping problem and preserves design efficiency.

## 2. Materials and Methods

**Figure 1:**
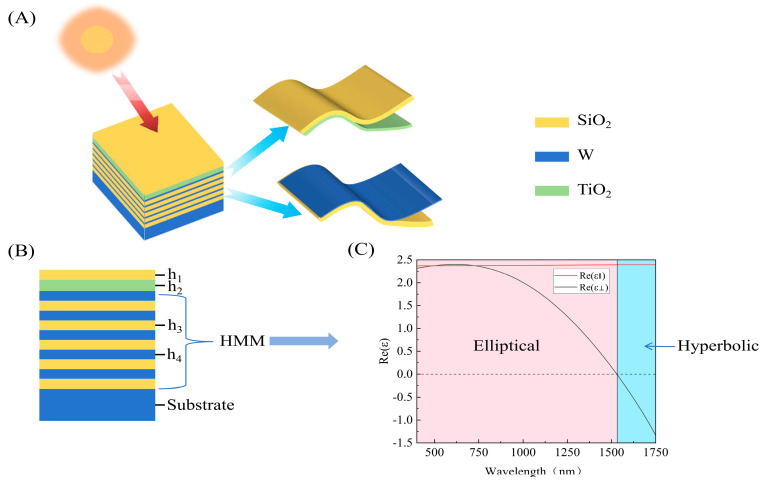
(**A**) 3D and (**B**) 2D schematic of the designed absorber. (**C**) The HMM structure with h_3_ = 112 nm, h_4_ = 13 nm. When Re(ℇ⟂)Re(ℇ∥) > 0, one can achieve an elliptical response; it turns into a hyperboloid when Re(ℇ⟂)Re(ℇ∥) < 0. The pink area highlights the spectral range of elliptical response. The blue area highlights the spectral range of hyperbolic response.

Our solar absorber adopts a multilayer architecture consisting of alternating W and SiO_2_ layers, capped with a TiO_2_/SiO_2_ bilayer, as depicted in Figure 1A. The W/SiO_2_ stack functions as a HMM, as shown in Figure 1B, supporting a high photonic density of states and enhanced in-plane wavevectors that promote broadband light trapping within the lossy W layers, as shown in Figure 1C. The top TiO_2_/SiO_2_ bilayer serves as an impedance-matching AR coating: by forming a graded refractive index transition from the air to the absorber, it effectively suppresses reflection across the solar spectrum.

**Figure 2:**
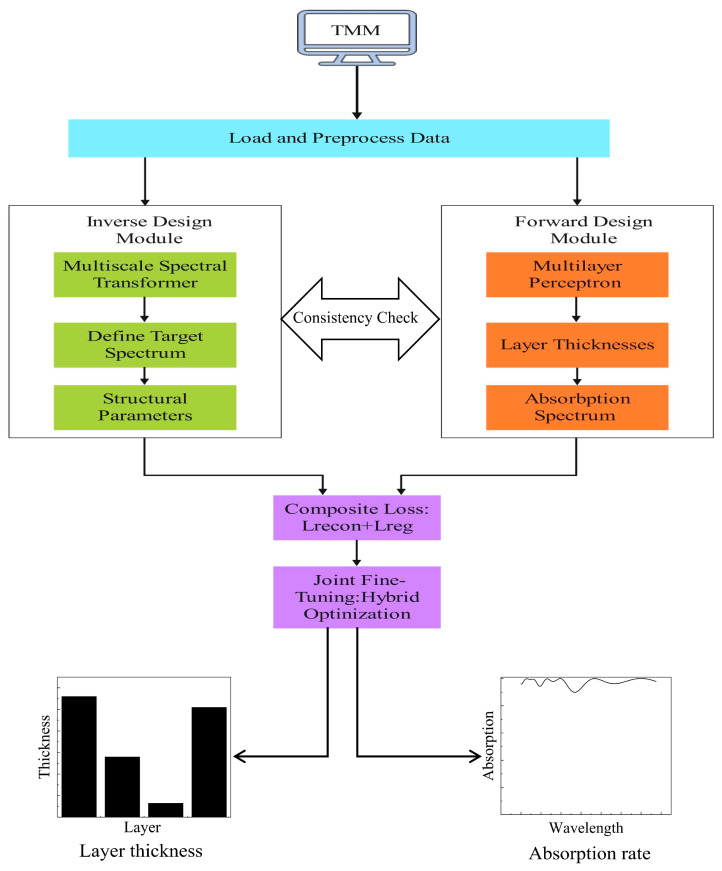
Deep learning-assisted joint training optimization scheme.

In order to optimize the absorber quickly and accurately, a joint optimization framework of a coupled forward–inverse design module based on an MST is proposed. As shown in Figure 2, the workflow first generates a large-scale data set containing 100,000 samples through a transfer matrix method (TMM) simulation implemented in MATLAB R2023b. Each sample has four geometric layer-thicknesses parameters and absorption spectra corresponding to 196 wavelengths between 400 and 1750 nm. StandardScaler is used to normalize the data set, and it is divided into a training set accounting for 90% and a test set accounting for 10%. Python 3.12 is used for the training.

The inverse design model of this framework uses the MST architecture to map the target absorption spectra to the structure parser. It applies a patch-based embedding strategy to handle the high-dimensional spectra input, dividing the 196-point spectra into 14 non-overlapping 14-point segments. Each segment is independently processed by a 1D convolution layer to generate the token embeddings. The position encoding retains the spec sequence information, and the resulting sequence enters a 12-layer Transformer encoder, which has 16-head self-attention mechanisms and a 2048-dimensional position-aware feed-forward network. The residual connections and layer normalization are used after each sub-layer to stabilize the training. The forward design module uses a 5-layer perceptron to map layer thicknesses to the spectral response. This module serves as a physical validity validator for the candidate designs proposed by the inverse module, forming a mutually complementary relationship with the MST-based inverse design module.

In our optimization framework, we adopt a three-stage training strategy to fully exploit the complementary nature of the branches and ensure physically consistent solutions. Initially, each branch is pre-trained independently for 500 epochs. Then, fine-tuning is carried out jointly for 800 epochs using the composite loss function $L={\alpha L}_{recon}+{\beta L}_{reg},\alpha =1,\beta =0.5$, where $L_{\mathrm{recon}}$ denotes the mean squared error (MSE) between the predicted and actual spectra, and $L_{\mathrm{reg}\;}$ represents the MSE between the predicted and actual parameters. This cyclic co-training paradigm allows the inverse model to propose candidate designs while the forward model validates their physical realizability, thereby mitigating the inherent one-to-many ambiguity in inverse design. The training stability and generalization are further enhanced through mixed-precision computation, ReduceLROnPlateau scheduling, early stopping, and spectral data augmentation via random noise injection.

The model performance is evaluated using the MSE between the predicted and TMM-simulated spectra. Quantitative comparisons confirm that the hybrid framework accurately captures the complex relationship between the geometric parameters and the broadband absorption response, enabling the rapid and reliable design of high-performance solar absorbers.

During all three training stages we apply training-only augmentation to standardized spectra: additive Gaussian noise ℇ ~ N (0, σ^2^) with σ = 0.02, followed by clipping to [−3, 3]. Augmentation is enabled for the training split only (augment = True), and is disabled for validation and testing. In addition, we adopt a dropout (*p* = 0.1–0.3), AdamW with weight decay = 1 × 10^−5^, ReduceLROnPlateau, and early stopping, which act as an implicit regularization to mitigate overfitting to the TMM-generated data.

## 3. Results and Discussion

### 3.1. Performance of the Proposed Optimization Framework

We comprehensively evaluate the performance of our joint optimization framework through a series of comparative theoretical analyses, assessing its forward prediction accuracy, inverse synthesis capabilities, and the advantages conferred by consistency-driven joint training. All the models are trained and tested on the same TMM-generated data set described in Section 2.

**Figure 3:**
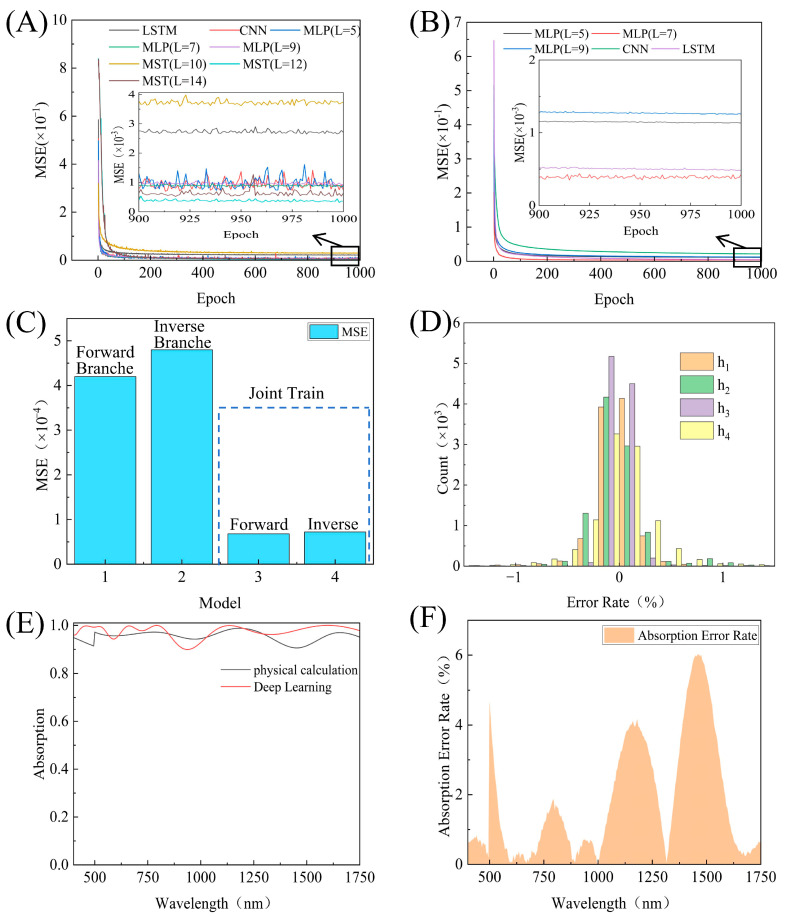
(**A**) Inverse model comparison (MST vs. MLP vs. LSTM vs. CNN). (**B**) Forward model comparison (MLP vs. LSTM vs. CNN). (**C**) Test MSE before vs. after joint training (forward and inverse). (**D**) Relative error distribution of predicted layer thicknesses. (**E**) Absorption spectra: DL prediction vs. physical calculation. (**F**) Wavelength-dependent spectral error rate between physical calculation and deep learning.

In the inverse design, where the target absorption spectra are mapped to the structural parameters, our MST significantly outperforms conventional architectures. As shown in Figure 3A, the MST in its optimized configuration of 12 transformer layers and 512-dimensional embeddings achieves a pre-joint-training test MSE of 4.8 × 10^−4^, substantially lower than those of a 7-layer MLP (7.2 × 10^−4^), a 6-layer LSTM (2.9 × 10^−3^), and an 8-layer CNN (6.8 × 10^−4^). For the forward design, which maps the four geometric parameters to the broadband absorption spectra, a compact 5-layer MLP proves highly effective. In the forward design, as illustrated in Figure 3B, the MLP attains a pre-joint-training test MSE of 4.2 × 10^−4^, outperforming deeper or more complex alternatives such as LSTM and CNN. This confirms that for this low-dimensional regression task, a simple MLP suffices to capture the underlying physics with high fidelity.

Figure 3C presents a comparison of the test MSE before and after joint training. The inverse model exhibits an order-of-magnitude reduction in error (7.2 × 10^−5^), and the forward model shows markedly improved spectral reconstruction accuracy (6.8 × 10^−5^). This confirms our hypothesis that when the consistency across directions is strong as a regularizer, it alleviates the ill-posedness of inverse design well.

The parameter level further confirms that the inversion prediction is relatively accurate. In Figure 3D, the relative errors of the predicted thickness of the four-layer clusters are distributed tightly around the zero value, and more than 90% of the sample errors are within ±1%. This narrow error distribution highlights the robustness and reliability of MST in actual inversion design.

The optimal structural parameters obtained through the joint optimization algorithm are summarized in Table 1. To evaluate the accuracy of the predicted spectra, we compare the absorption spectrum derived from the structural optimization prediction with the result of physical calculation. As shown in Figure 3E, the two spectra exhibit excellent agreement across the 400–1750 nm wavelength range. The wavelength-resolved error rate (Figure 3F) remains below 5% over most of the band, with minor deviations in the near-infrared region—likely due to reduced spectral sensitivity in that range.

The relief of one-to-many ambiguity is achieved by quantifying the joint training. The statistics show the stability and consistency of inverse prediction. We evaluate three indicators: the parameter Mean Absolute Error (MAE), the compactness of solution distribution (using covariance trace), and the forward–inverse cycle consistency error (Cycle MSE). More than 80% of the test samples in Table 2 reach the preset thresholds for all indicators. The low covariance trajectory values show that the predicted structural parameters are clustered in a narrow manifold, indicating that the proposed joint training can effectively suppress the ambiguity. The low-period MSE proves the strong forward–backward consistency, meaning that the predicted structure can reliably reproduce the target spectrum. The cycle consistency makes the joint forward–inverse design very reliable at the physical level.

To verify the advantages of the proposed framework, benchmark tests are carried out with a standard Tandem Network on the same multilayer absorber system. In the series architecture, first train the forward MLP and then fix it; the inverse model is optimized by relying on the spectral reconstruction loss of freezing the forward predictor. This method uses cyclic consistency regularization to carry out joint forward–inverse training. Both models are evaluated on the same TMM test set using the spectral accuracy, parameter accuracy, design yield, and average inference time. The design yield is the proportion of predicted designs where the spectral MSE is below the set threshold. The results are shown in Table 3. The joint forward–inverse model signal outputs are lower than all the error metrics of the series-connected network. When the spectral MSE threshold is 1.0 × 10^−3^, the yield of the joint framework is 93.16%, and the series-connected network cannot obtain an effective density estimation. When using a stricter threshold of 5.0 × 10^−4^, the output rate of the combined model still remains at 83.52%. The accuracy and yield are significantly improved, while the average inference time is roughly similar (1.68 ms and 1.75 ms), which indicates that the performance improvement is achieved through consistent-driven training, not due to higher computational costs. These results show that the proposed joint optimization strategy is stable, practical and effective.

### 3.2. Performance Evaluation of Absorbers

To elucidate the physical origin of the broadband near-perfect absorption achieved by the proposed absorber, we perform full-wave finite-difference time-domain (FDTD) simulations to analyze the spatial distribution of electromagnetic energy and its dissipation pathways.

**Figure 4:**
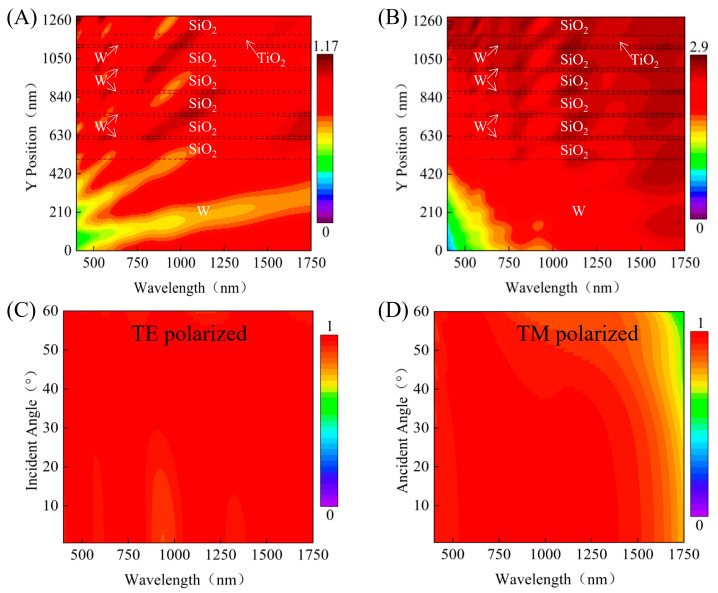
(**A**) Simulated contour map of the electric field intensity distribution, |E|. (**B**) Corresponding contour map of the power absorption density, Pabs. (**C**) Angle-resolved absorptance spectra under TE polarized. (**D**) Angle-resolved absorptance spectra under TM polarized.

Figure 4A,B show the wavelength-dependent |E| and P_abs_ in the cross-section of structure X–Z. In the visible wavelength range (400–700 nm), the strong fields are confined near the air interface in the upper W/SiO_2_ layer due to the constructive interference and resonant modes excited by the top anti-reflection coating. When the wavelength reaches the near-infrared region (700–1750 nm), the electromagnetic energy penetrates deeper, and the field is enhanced at specific W/SiO_2_ interfaces. The |E| gradually attenuates along the propagation direction, indicating that energy accumulates and dissipates in the stacked structure.

This behavior conforms to the formula $\mathrm{Pabs}\;=\frac{1}{2}\omega \varepsilon^{\prime \prime }{\left|E\right|}^{2}$, where $\varepsilon^{\prime \prime }$ is the imaginary part of the dielectric constant and ω is the angular frequency. In Figure 4B, the W layer with a larger imaginary part of the dielectric constant is the main optical loss point, which converts light into heat. The W layer with short wavelengths at the top is absorbed, and long wavelengths are dissipated by the deeper W layer. The SiO_2_ spacer layer realizes broadbanding by adjusting the interlayer interface and field distribution. The top TiO_2_/SiO_2_ double-layer film is an impedance-matching AR film, which can minimize the surface reflection. The thicker bottom W layer is an ideal back reflector, which can eliminate transmission and maintain an absorption rate close to unity.

Figure 4C,D show the absorption spectra under TE and TM polarizations for incident angles from 0 to 60. For TE polarization, the range with an absorption efficiency exceeding 90% at 20° is 404–1465 nm (bandwidth of 1061 nm), and at 60° it is still 400–1024 nm (bandwidth of 624 nm). The performance of TM polarization is more stable: the range with absorption exceeding 90% at 20° is 400–1134 nm (bandwidth of 734 nm), and at 60° it extends to 400–1358 nm (bandwidth of 958 nm). The polarization insensitivity and wide-angle response originate from the isotropic planar geometry, and there is no need for lateral patterning.

**Figure 5:**
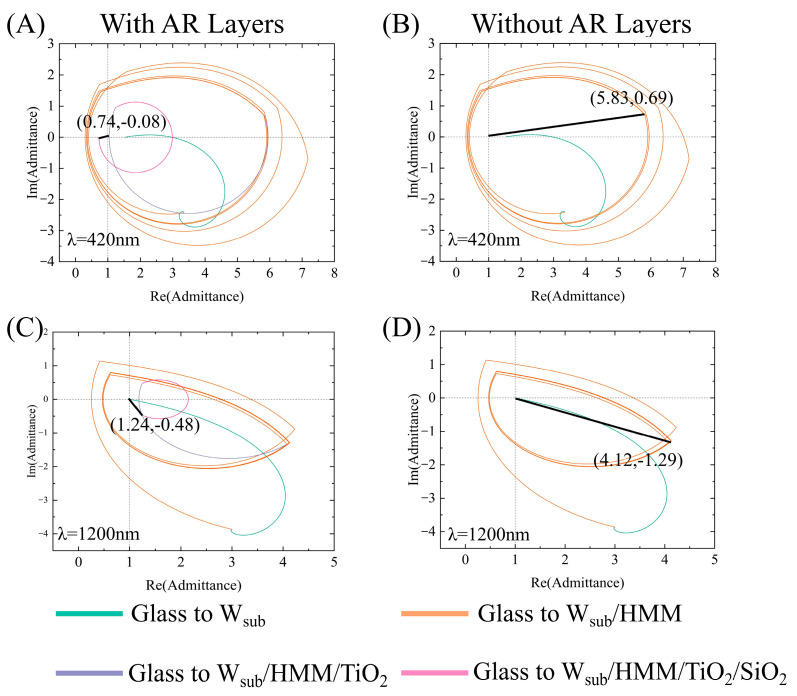
(**A**) Admittance loci at 420 nm with AR layers. (**B**) Admittance loci at 420 nm without AR layers. (**C**) Admittance loci at 1200 nm with AR layers. (**D**) Admittance loci at 1200 nm without AR layers. The black line in each subfigure indicates the final admittance matching trajectory toward free-space admittance, highlighting the impedance-matching condition at the corresponding wavelength.

The role of the TiO_2_/SiO_2_ bilayer in achieving broadband AR is further quantified using optical admittance locus theory. The locus starts from the substrate point (nsub, 0), which is the reflective tungsten back layer, and spirally extends through the W/SiO_2_ stacked structure. The best matching occurs when the terminal admittance of the complete structure converges to the air point (1, 0) to achieve the minimum reflectivity. Figure 5 presents the admittance loci diagrams of the structure with and without the AR layers at different wavelengths, including the values of 420 nm and 1200 nm. The AR structure locus is closer to (1, 0), which means better impedance matching in the solar spectrum. This directly explains the suppression reflectivity and also lays the foundation for the observed absorption rate of nearly 100% in numerical simulation [28].

### 3.3. Outdoor Performance Testing

**Figure 6:**
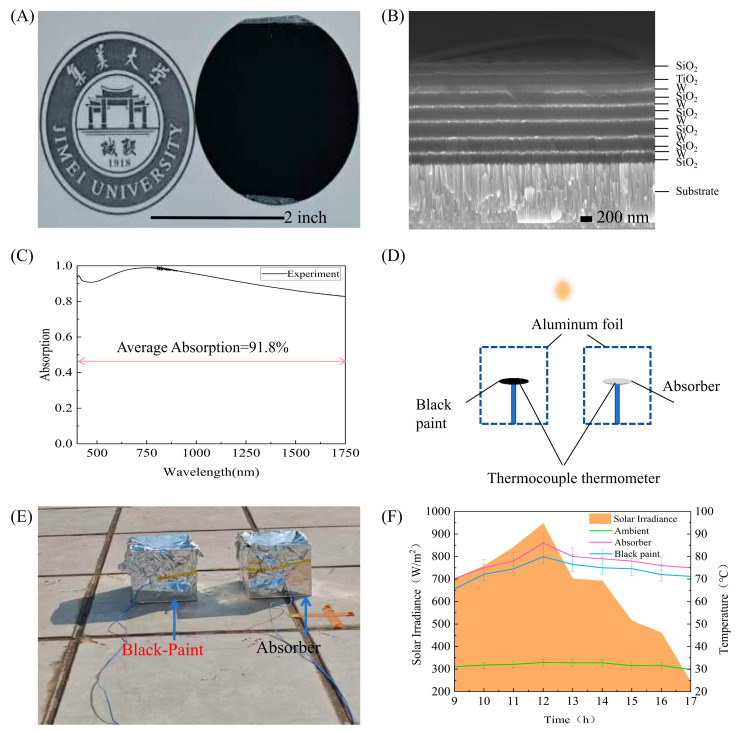
(**A**) Photograph of the fabricated absorber (2-inch diameter). (**B**) Cross-sectional SEM image of the fabricated absorber. (**C**) Experimental absorption spectra of the designed absorber at normal incidence. (**D**) Schematic and (**E**) photograph of the outdoor experimental setup for photothermal testing under natural sunlight. (**F**) Temporal profiles from 9:00 to 17:00 of solar irradiance (shaded orange) and surface temperatures of the absorber (pink), black-painted reference (blue), and ambient air (green). Error bars represent the standard deviation obtained from repeated measurements at each time point.

Building upon the theoretical design, we fabricated and experimentally validated the proposed solar absorber. The absorber was deposited on a 2-inch-diameter silicon wafer using a high-vacuum electron-beam evaporation system (DZS-500, Shenyang Scientific Instruments Co., Ltd., Chinese Academy of Sciences, Shenyang, China). As shown in Figure 6A, the resulting wafer-scale sample exhibits a uniform visual appearance, confirming large-area process compatibility.

To verify whether the fabricated absorber matches the designed structure, a cross-sectional scanning electron microscope (SEM) image was acquired, as shown in Figure 6B. The image clearly reveals the W and SiO_2_ layers, as well as the top TiO_2_/SiO_2_ bilayer structure, which confirm the successful fabrication of the intended multilayer architecture. Figure 6C presents the average absorption spectrum of the fabricated structure. It is shown that the average absorption rate of this structure reaches 91.8%. This value deviates slightly from the physically simulated result. We explored the difference between the simulated average absorption rate (97.4%) and the experimentally measured value (91.8%), which was obtained using a UV–Vis–NIR spectrophotometer (Cary 5000, Agilent Technologies, Santa Clara, CA, USA), and carried out a quantitative sensitivity analysis to evaluate the impact of uncertainties caused by actual fabrication factors on the optical performance. Table 4 shows the average absorption for ±5% variations in the structural parameters and refractive indices. The results show that: (1) When the single-layer thickness varies by ±5%, the average absorption rate decreases by about 1% to 3%. (2) When the refractive index of SiO_2_ changes by ±5%, the absorption changes by about 4%. There is a similar sensitivity to the refractive index deviation of TiO_2_. Small reasonable variations in the optical constants and thickness parameters can explain the adsorption rate dropping from 97.4% to about 91.8%. 

Outdoor experiments were conducted on the rooftop of the Keli Building at Jimei University in Xiamen, Fujian Province, China, under natural light to evaluate the photothermal conversion performance. Figure 6D,E show a schematic illustration and photographs, respectively, of the heating measurement system used in the experiments. The surface temperatures were measured using a NI-T325K thermocouple (produced by Uni-Trend Technology (China) Co., Ltd., Dongguan, China). In this study, the photothermal performance of the fabricated absorber was directly compared with that of a conventional black-painted film under identical outdoor conditions. The results are shown in Figure 6F. Figure 6F shows the solar irradiance (orange) and surface temperature from 9:00 to 17:00. To handle the uncertain factors in the outdoor measurement environment (such as wind speed, convective heat loss, short-term radiation fluctuations, etc.), it was necessary to repeat the temperature measurement at each time point. The error bars in Figure 6F are the standard deviations of the measured surface temperatures, which were used to measure the experimental uncertainties. The irradiance gradually increased after 9:00, reached a peak of about 850 W/m^2^ at noon, and then decreased. The ambient temperature (green line) during the test stabilized between 30℃ and 33℃. The absorber with the pink curve tracked the solar irradiance, and the temperature was close to 86.3 °C at noon. Under the same conditions, the traditional black-painted reference surface (blue curve) reached about 80 °C. This increase of about 6.3 °C shows the advantages of this absorber in terms of its solar absorption capacity and photothermal conversion efficiency in practical applications.

## 4. Conclusions

This work constructs a joint forward–inverse DL framework to solve the ill-posed inverse design problem in broadband solar-absorbing metamaterials. End-to-end consistency is achieved between the inverse model based on an MST and the forward model based on a MLP, and the high-precision and stable optimization of multilayer absorber structures is realized. The proposed framework yields a normalized MSE of 7.2 × 10^−5^ for the inverse prediction and 6.8 × 10^−5^ for the forward prediction on the test set, demonstrating a substantial improvement in the inverse design fidelity. Using this approach, a broadband absorber composed of W/SiO_2_ HMM stacks integrated with TiO_2_/SiO_2_ AR coatings is optimized, exhibiting an average absorption of 97.4% over the 400–1750 nm solar spectrum, with polarization insensitivity and robust wide-angle performance up to 60°. The device’s fabrication and outdoor testing further validate the practical feasibility of the design, achieving a peak temperature of 86.3 °C under natural sunlight, with an irradiance peak of about 850 W/m^2^ at noon. These results demonstrate that consistency-driven forward–inverse DL provides an efficient and scalable paradigm for the intelligent design of high-performance photothermal metamaterials, with strong potential for extension to multiobjective spectral and energy-conversion applications.

## Figures and Tables

**Table 1:** Optimal structural parameters of the multilayer solar absorber.

Stack Order (Top > Bottom)	Material	Thickness (nm)
h_1_	SiO_2_	102
h_2_	TiO_2_	56
h_3_	W	13
h_4_	SiO_2_	112

**Table 2:** Statistical summary of parameter error, uncertainty, and cycle consistency (10,000 test samples).

Metric	Parameter MAE	Covariance Trace	Cycle MSE
Mean	3.9 × 10^−2^	7.2 × 10^−2^	1.3 × 10^−1^
Median	2.9 × 10^−2^	6.5 × 10^−2^	9 × 10^−2^
Min	1 × 10^−3^	9 × 10^−3^	6 × 10^−5^
Max	1.296	1.397	2.667
Threshold	<5 × 10^−2^	<1 × 10^−2^	<1.5 × 10^−2^
Percentage of Samples	80.6%	83.7%	84.2%

**Table 3:** Performance comparison of the joint forward–inverse and Tandem neural network strategies for inverse design of the target material system, in terms of accuracy, design yield, and inference time.

Method	Joint Forward–Inverse	Tandem Network
Spectral_MSE	5.7 × 10^−5^	2.6 × 10^−2^
Spectral_MAE	1.3 × 10^−3^	1.4 × 10^−2^
Param_MSE	7.9 × 10^−5^	2.5 × 10^−2^
Param_MAE	5.6 × 10^−3^	1.1 × 10^−2^
Avg_Inference_Time_ms	1.68 ms	1.75 ms
Percentage of Yield_MSE < 1.0 × 10^−3^	93.16%	0
Percentage of Yield_MSE < 5.0 × 10^−4^	83.52%	0

**Table 4:** Sensitivity analysis of average absorption for ±5% variations in structural parameters and refractive indices.

Modify Parameters	Increase by 5%	Reduce by 5%
h1 thickness	94.2%	93.8%
h2 thickness	93.0%	95.7%
h3 thickness	91.5%	92.0%
h4 thickness	93.0%	93.3%
SiO_2_ refractive index	92.5%	96.4%
TiO_2_ refractive index	95.3%	95.4%

## Data Availability

Data are available on request from the corresponding authors.

## Abbreviations

The following abbreviations are used in this manuscript:

HMMs	Hyperbolic Metamaterials
DL	Deep Learning
LSTM	Long Short-Term Memory
ANN	Artificial Neural Network
CGAN	Conditional Generative Adversarial Network
AR	Anti-Reflective
MST	Modified Swin Transformer
MLP	Multilayer Perceptron
TMM	Transfer Matrix Method
MSE	Mean Squared Error
FDTD	Finite-Difference Time-Domain
SEM	Scanning Electron Microscope
MAE	Mean Absolute Error

## References

[1] Liu F., Lai Y., Zhao B., Bradley R., Wu W. (2019). Photothermal materials for efficient solar powered steam generation. Front. Chem. Sci. Eng..

[2] Gao M., Zhu L., Peh C.K., Ho G.W. (2019). Solar absorber material and system designs for photothermal water vaporization towards clean water and energy production. Energy Environ. Sci..

[3] Wu X., Chen G.Y., Owens G., Chu D., Xu H. (2019). Photothermal materials: A key platform enabling highly efficient water evaporation driven by solar energy. Mater. Today Energy.

[4] Xu K., Du M., Hao L., Mi J., Yu Q., Li S. (2020). A review of high-temperature selective absorbing coatings for solar thermal applications. J. Mater..

[5] Yuan K., Chen B., Shan S., Xu J., Yang Q. (2024). A high-temperature solar selective absorber based on one-dimensional multilayer nanostructures. Sol. Energy Mater. Sol. Cells.

[6] Rephaeli E., Fan S. (2008). Tungsten black absorber for solar light with wide angular operation range. Appl. Phys. Lett..

[7] Wu D., Liu C., Liu Y., Xu Z., Yu Z., Yu L., Ye H. (2018). Numerical study of a wide-angle polarization-independent ultra-broadband efficient selective metamaterial absorber for near-ideal solar thermal energy conversion. RSC Adv..

[8] Cai H., Wang M., Wu Z., Wang X., Liu J. (2022). Design of multilayer planar film structures for near-perfect absorption in the visible to near-infrared. Opt. Express.

[9] Chen Q., Gu J., Liu P., Xie J., Wang J., Liu Y., Zhu W. (2018). Nanowire-based ultra-wideband absorber for visible and ultraviolet light. Opt. Laser Technol..

[10] Li Y., Liu Z., Zhang H., Tang P., Wu B., Liu G. (2019). Ultra-broadband perfect absorber utilizing refractory materials in metal-insulator composite multilayer stacks. Opt. Express.

[11] Yuan H., Yi Y., Song Q., Yi Z., Sun T., Tang C., Zeng Q., Cheng S., Wu P. (2024). Ultra-broadband absorber and perfect thermal emitter for high-efficiency solar energy absorption and conversion. Renew. Energy.

[12] Yuan X., Wang Q., Kuang K., Gao H., Liang Y., Peng W. (2023). Lithography-free near-infrared broadband absorber based on a multilayer nanosystem. Optik.

[13] Ding F., Mo L., Zhu J., He S. (2015). Lithography-free, broadband, omnidirectional, and polarization-insensitive thin optical absorber. Appl. Phys. Lett..

[14] Ma Y., Hu J., Li W., Yang Z. (2023). Angle-insensitive ultrathin broadband visible absorber based on dielectric–semiconductor–lossy metal film stacks. Nanomaterials.

[15] Abedini Dereshgi S., Ghobadi A., Hajian H., Butun B., Ozbay E. (2017). Ultra-broadband, lithography-free, and large-scale compatible perfect absorbers: The optimum choice of metal layers in metal-insulator multilayer stacks. Sci. Rep..

[16] Prikaen C., Chaisakul P., Chiangga S. (2024). Design and optimization of broadband near-perfect absorber based on transition metal nitrides thin-films for solar energy harvesting. Results Phys..

[17] Seo J., Jung P.H., Kim M., Yang S., Lee I., Lee J., Lee B.J. (2019). Design of a broadband solar thermal absorber using a deep neural network and experimental demonstration of its performance. Sci. Rep..

[18] Gai Y., Zhou S., Lan G. (2025). Optimization of broadband solar metamaterial absorber based on deep neural network. Plasmonics.

[19] Almawgani A.H., Sorathiya V., Soni U., Alhawari A.R., Daher M.G. (2024). Numerical investigation of MXene-based ultrawideband solar absorber with behaviour prediction using machine learning. Opt. Quantum Electron..

[20] Cerniauskas G., Sadia H., Alam P. (2024). Machine intelligence in metamaterials design: A review. Oxf. Open Mater. Sci..

[21] Ding Z., Su W., Ye L., Li W., Zhou Y., Tang B., Zou J., Yao H. (2024). Deep learning based inverse design of metasurface absorber for maximizing solar spectral absorption. Sol. Energy.

[22] Xu J., Xu P., Yang Z., Liu F., Xu L., Lou J., Fang B., Jing X. (2024). Freeform metasurface design with a conditional generative adversarial network. Appl. Phys. A.

[23] Ma T., Ma M., Guo L.J. (2025). Optical multilayer thin film structure inverse design: From optimization to deep learning. iScience.

[24] Guan Q., Alketbi A.S., Raza A., Zhang T. (2020). Accelerated development of refractory nanocomposite solar absorbers using Bayesian optimization. MRS Adv..

[25] Ma T., Wang H., Guo L.J. (2023). OptoGPT: A foundation model for inverse design in optical multilayer thin film structures. arXiv.

[26] Jenkins R.P., Campbell S.D., Werner D.H. (2021). Establishing exhaustive metasurface robustness against fabrication uncertainties through deep learning. Nanophotonics.

[27] Ueno A., Lin H.I., Yang F., An S., Martin-Monier L., Shalaginov M.Y., Hu J. (2023). Dual-band optical collimator based on deep-learning designed, fabrication-friendly metasurfaces. Nanophotonics.

[28] Zhang H., Fan D.W., Yu T.Z., Wang C.L. (2015). Antireflective and self-cleaning properties of SiO_2_/TiO_2_ double-layer films prepared by cost-effective sol-gel process. Chin. J. Chem. Phys..

